# Assessing Predictors of Changes in Protein Stability upon Mutation Using Self-Consistency

**DOI:** 10.1371/journal.pone.0046084

**Published:** 2012-10-29

**Authors:** Grant Thiltgen, Richard A. Goldstein

**Affiliations:** Department of Mathematical Biology, National Institute for Medical Research, Mill Hill, London, United Kingdom; University Of Oxford, United Kingdom

## Abstract

The ability to predict the effect of mutations on protein stability is important for a wide range of tasks, from protein engineering to assessing the impact of SNPs to understanding basic protein biophysics. A number of methods have been developed that make these predictions, but assessing the accuracy of these tools is difficult given the limitations and inconsistencies of the experimental data. We evaluate four different methods based on the ability of these methods to generate consistent results for forward and back mutations, and examine how this ability varies with the nature and location of the mutation. We find that, while one method seems to outperform the others, the ability of these methods to make accurate predictions is limited.

## Introduction

The stability of a protein is generally represented by the change in the Gibbs free energy upon folding (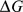), where an increasingly negative number represents greater stability. The substitution of a single amino acid in a protein sequence can result in a significant change in the protein's stability (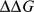), where a positive 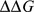 represents a destabilizing mutation and a negative value represents a stabilizing mutation. The ability to understand and to predict the size and magnitude of these changes is an important goal for a number of different reasons. Firstly, we are often interested in modifying proteins in order to provide them with specific properties such as enhanced stability. Given the number of possible mutations, it is critical for us to be able to predict which ones are likely to have the desired effect. Secondly, we are often interested in understanding the physiological effect of various single nucleotide polymorphisms (SNPs) that are found in some fraction of the population. We might expect that SNPs that result in a significant change in protein properties are more likely to be deleterious. Thirdly, understanding of how substitutions affect protein properties are an essential part of the program to connect protein biophysics and evolutionary analyses. Maybe most broadly, being able to predict the impact of a substitution on a protein's property is a way of refining our understanding of the general principles of protein thermodynamics.

To satisfy these goals, a number of programs have been developed that estimate the effect of a mutation on the stability of a protein, using either biophysical models of amino acid interactions [1]–[4], statistical analyses of available proteins and their thermodynamic properties [5]–[7], machine learning methods [8], [9], or a combination thereof [10], [11]. With the availability of these programs comes the need for them to be evaluated and compared. The most straightforward approach is to compare the 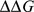 predictions generated by these programs to experimental data, such as those compiled in the ProTherm [12] database. Recently, there have been two independent comparisons of these 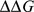 predictors. The first comparison used a set of 2156 mutations from the ProTherm database in order to compare six different methods for 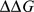 predictions: FoldX [5], CC/PBSA [13], Rosetta [14], EGAD [15], I-Mutant2.0 [16], and Hunter [17]. EGAD performed best with a correlation coefficient of 0.59, while Rosetta performed the worst in this evaluation with a correlation coefficient of 0.26. The range of coefficients for the other five methods ranged from 0.45 to 0.59, indicating roughly similar performance. One limitation of this study was that the metric used for assaying performance, the correlation coefficient between computed and experimentally determined values, is insensitive to systematic biases - a method that predicts values of 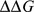 that are too high by a constant 10 kcal/mol, or underestimates these values by a constant factor of 1/2, could still have a perfect correlation coefficient of 1.0.

A second study of eleven predictors also compared their computed values to values from ProTherm, but rather than using the correlation coefficient the methods were evaluated based on their ability to classify mutations into stabilizing mutations (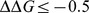), destabilizing mutations (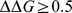), and neutral mutations (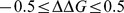) [18]. Their comparison showed I-Mutant3.0 [8] to be the most accurate predictor for the three state prediction.

A limitation of all of these methods that compare predicted versus measured changes in stability is variability of 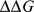 values in the database. The value of 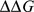 can depend upon the experimental method used as well as the temperature, pH, ionic strength, presence of denaturants, redox state of co-factors, method of protein preparation, etc. Thus comparing 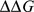 values calculated using different experimental methods may create confusion when creating datasets for training. One mutation (C112S in Pseudomonas aeruginosa azurin, PDB 5AZU), for example, occurs twelve times in the ProTherm database with 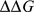 values ranging from 0.24 to 4.40 kcal/mol [19]. It is not clear what are the experimental conditions that correspond with the methods used for making the predictions. This has lead some investigators to simply use the average of the values for each mutation [1], [19], an unsatisfactory solution that makes the comparisons dependent upon the distribution of experiments included in the database.

Because of the variability of experimental results and the difficulty of determining which of these values should be used as the “correct” value of 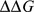, we propose a new type of evaluation. We would expect that, whatever experimental conditions are most appropriate match for the calculations, these calculations should themselves be self-consistent. In particular, mutating a given location from *X* to *Y* should have an opposite effect to the reverse mutation from *Y* to *X*, that is, 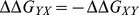. This exact equality will not be satisfied by available prediction methods due to the limitations, heuristics, and approximations that these methods necessarily make. It does, however, provide a standard with which prediction methods can be compared, providing an estimate for the accuracy of these methods.

We make this comparison by finding 65 pairs of protein with known crystal structures, where the members of each pair differ at only a single location. We can then consider mutations in each protein so that the mutant protein matches the other protein in the pair. We propose checking this consistency rather than comparing to experimental values that may or may not be accurate. In particular, by making a few modest assumptions, we can estimate and compare the magnitude of the errors of different computational methods without requiring any information about the real values of 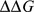.

We tested this method of evaluating predictors by applying it to four different methods for calculating 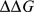: FoldX [5], Rosetta's ddg_monomer method [1], the Eris web-server [2], and I-Mutant3.0 [8]. While Rosetta has been evaluated in one of the two previous comparisons, their new method incorporating a flexible backbone had not been tested. The Eris method was also not evaluated by either of the two previous comparisons, and it is also a method that allows for a flexible backbone. We find that Rosetta provides, in general, more accurate results than the other three methods.

## Results

### Comparison of methods

**Figure 1:**
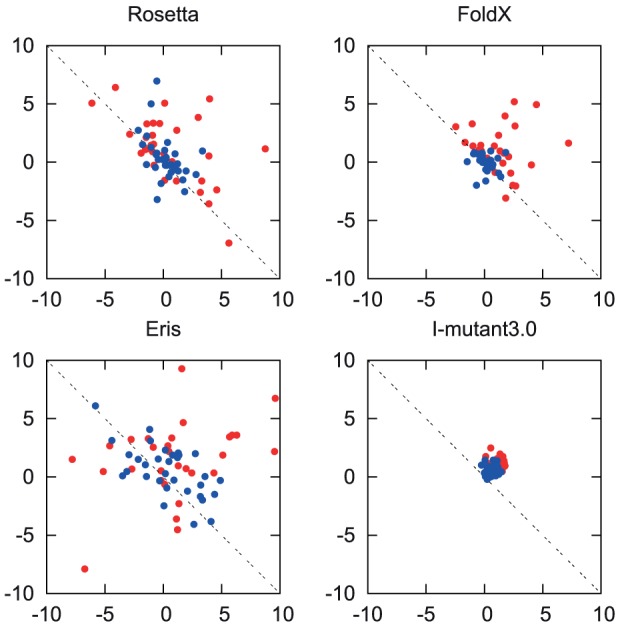
A scatter diagram of 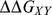 against 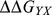. Values are in kcal/mol. The blue dots represent the exposed set of the mutations (relative solvent accessibility 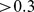) and the red dots represent the buried set. The dotted lines represent the expectation that 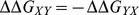.

Calculated predictions of 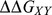 and 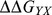 are shown for the four methods in Figure 1. We find that there is a significant discrepency between the predictions by all three methods and the expectation of 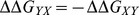. The exact values of 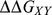 and 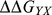 for each method can be found in Table S1.

**Figure 2:**
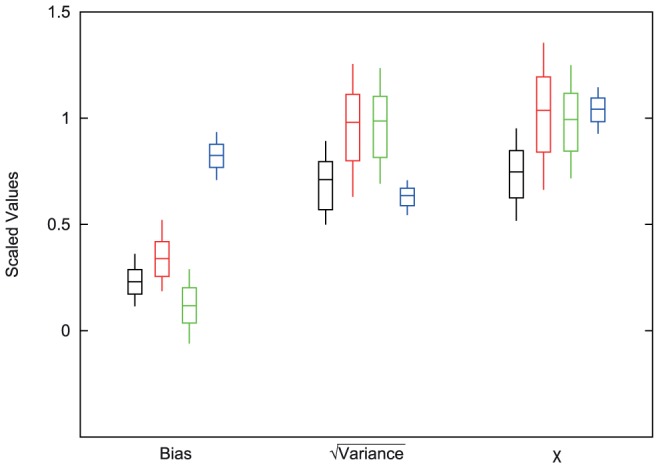
A comparison of the methods for bias, 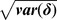, and 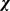 scaled by RMS of the predictions. The center bars represent the calculated value for each of the methods. The top and bottom bars represent the 67% confidence intervals and the thin vertical lines extend to the 95% confidence intervals. The order of methods is Rosetta (black), FoldX (red), Eris (green), and iMutant3.0 (blue). For Rosetta, FoldX, and Eris the contributing factor for 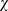 appears to be the Variance, while I-Mutant3.0 seems to be affected more by the bias.

One limitation of the evaluation method is that it is unable to determine the scale of the predicted values, yet this scaling would also scale the estimated errors; the estimated error would be reduced, for instance, by multiplying all of the calculated estimates of 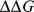 by a number smaller than one. The four methods generate predictions with significantly different magnitudes, with the RMS of the predicted 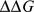 values equal to 0.97, 1.58, 2.41, and 3.95 for I-Mutant3.0, FoldX, Rosetta, and Eris, respectively. To counteract this bias, we scaled the calculated errors by the root mean square (RMS) of the predicted values for each method. We estimated the systematic biases in the computational predictions of changes in thermodynamic stability, as well as the variance of the random component of the error, where this random component has mean zero. The scaled systematic bias as well as the scaled square root of the variance in the error for each method (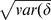) are shown in Figure 2. Also shown is the RMS of the error (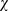, calculated with Equation 8), again scaled by the RMS of the predictions. We find that Eris has the smallest systematic bias, while the bias of I-Mutant3.0 is substantially higher than that of the other methods. The random component of the error is smaller for Rosetta and I-Mutant3.0. Overall, Rosetta has significantly lower errors (as characterized by 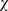) compared with the other three methods (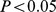).

We can characterize the absolute performance of these three methods by estimating the fraction of the variation explained by the predictions by calculating one minus the ratio of the variance in the error divided by the variance in the computed values. The results are not pleasing, with values of 0.44 for Rosetta and essentially zero for the other methods.

### Comparison of mutation types

**Figure 3:**
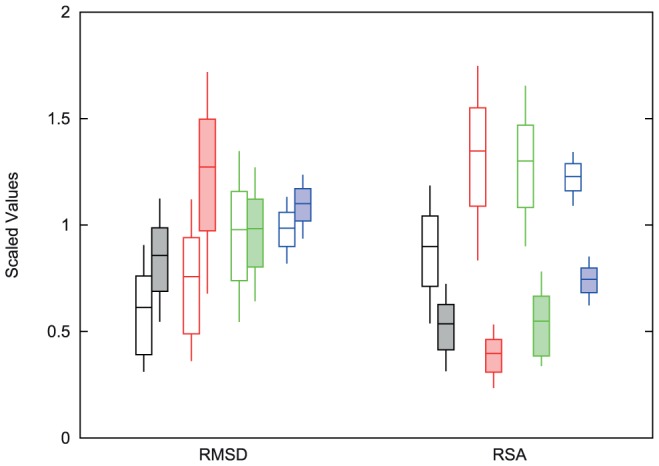
A comparison of the 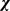 value with the RMSD datasets and RSA datasets scaled by RMS of the predictions. The center bars represent the calculated value for each of the methods. The top and bottom bars represent the 67% confidence intervals and the thin vertical lines extend to the 95% confidence intervals. The order of methods is Rosetta (black), FoldX (red), Eris (green) and iMutant3.0 (blue). The open RMSD bars represent those pairs of proteins with small changes in the two structures (RMSD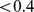) and the shaded bars represent the pairs with larger changes. The open RSA bars represent those mutations that are buried within the protein (RSA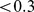) and the shaded bars are those mutations that are more exposed. The RMSD split shows that Rosetta and I-Mutant3.0 do slightly better on structures with a lower RMSD value, while Eris performs equally as well on both sets. FoldX shows the most change between these two protein sets. All the methods perform better on exposed mutations than buried mutations, with Rosetta doing the best on buried and FoldX doing the best on exposed.

In order to better characterize the performance of these various predictors, we categorized the mutations in two ways; either how conservative the mutation was in terms of the effect on the protein structure, as measured by root mean square deviation (RMSD) between the two protein structures, or where the mutation was relative to the surface of the protein, as indicated by relative solvent accessibility (RSA). Figure 3 shows the estimated accuracy (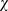 scaled by the RMS of the predictions) of these computational methods for structure conserving (RMSD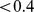), structure changing (RMSD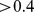), surface (RSA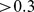), and buried (RSA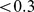) mutations. Separate values for the systematic bias and random error are shown in Figures S1 and S2.

No method showed a significant difference between the accuracy obtained with structure conserving and structure changing mutations, although FoldX, which assumes a fixed backbone, was close (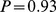). Eris showed the smallest dependence on the amount of structural change, with an increased bias for structure changing mutations countered by a decrease in the random error. All four methods exhibited substantially better results with mutations at exposed sites compared with buried sites. Interestingly the systematic bias was higher for Rosetta, FoldX, and I-Mutant3.0, while the random error was substantially higher for Rosetta, FoldX, and Eris. For all categories, Rosetta was as good as or superior to the other methods, with the exception of mutations at surface locations, where FoldX was slightly (although not statistically significantly) better.

## Discussion

We present an evaluation for 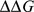 predictors that avoids the use of inconsistent experimental values. By making a limited set of assumptions involving the statistical properties of the errors, we are able to characterize the errors of the predictions by considering pairs of proteins of known structure, separated by a single mutation. Unlike approaches which consider correlation coefficients between predictions and actual values [17], we can characterize the systematic bias and the random errors separately. Similarly to those methods, however, we have difficulties with systematic scaling of the values; if all of the values were multiplied by a constant, we would not be able to detect the resulting discrepency. Possibly more seriously, the estimated errors scale with this constant. We can account for this effect by scaling the different errors by the RMS of the predicted values.

By considering the results with the scaled data, it appears that Rosetta performs the best of the three methods evaluated, with FoldX, Eris, and I-Mutant3.0 performing somewhat worse. In particular, the much smaller random errors achieved by I-Mutant3.0 were countered by a much higher systematic bias, approximately the same magnitude as the values of the predictions. The observed bias may represent the machine-learning techniques used by I-Mutant3.0, in that the database of mutations may be weighted towards destabilizing mutations. The various methods were generally insensitive to the amount of structural change involved by the mutation, measured by the RMSD of the two protein structures, with the possible exception of FoldX, which employs biophysical approaches assuming a fixed protein backbone. All four methods did substantially worse with buried locations, as would be expected due to the complexity of the local environment.

Our analysis assumes that the distributions of errors for the forward and reverse mutations are similar. This is a reasonable assumption for the biophysical methods such as Rosetta and Eris, but machine learning approaches such as I-Mutant3.0 may be better at predicting mutations away from the wild type than reverse mutations to the wild type [11], as the forward mutations may be more frequent in the training sets. If our assumption is incorrect, it will still be true that the bias will reflect the average bias of the forward and reverse mutation, and the variance will be 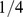 the sum of the variances for these two mutations. For some applications it may be better for the errors to be smaller for the forward mutation, especially when considering whether a SNP is deleterious. For understanding the relationship between phenotypic change and (generally reversible) evolutionary processes, or understanding the fundamentals of protein biophysics, however, there is a need to make accurate predictions in both directions. And when considering the needs for protein engineering, we are particularly interested in stabilizing mutations which may correspond more closely to reverse mutations.

The results demonstrate that there is much work that needs to be done to improve 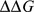 predictions, especially for buried amino acids, with Rosetta the only program that can explain a significant fraction of the observed variance in 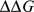 values.

## Methods

### Model

Consider a mutation at a given location from amino acid *X* to amino acid *Y*, and the corresponding back mutation from amino acid *Y* to amino acid *X*, where 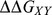 and 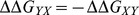 are the true but unknown changes in stability for these two mutations, respectively. These quantities are predicted by our computational model to have the values 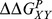 and 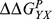, respectively, resulting in errors 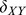 and 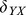:
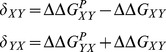
(1)


We do not know the correct value of 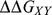. We instead consider, initially, the value (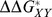) that would minimize the error, given by.
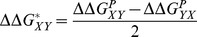
(2)


We can also consider 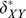 and 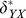, the values of 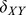 and 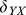 that would result if 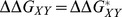:
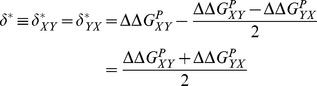
(3)where we have used the equality of 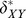 and 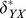 to define 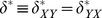. By combining equations 1 and 3, we get
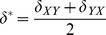
(4)


The distribution of errors 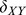 and 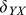 produced by the computational method can be characterized by a systematic bias 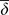, as well as a random component with mean 0 and variance 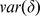. These parameters can be calculated by considering the averages and variances of both sides of equation 4, resulting in
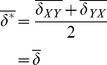
(5)and
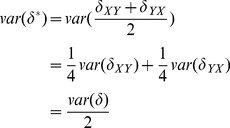
(6)where we have taken advantage of the fact that the designations of *X* and *Y* are arbitrary, so that the variance and bias of 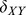 and 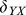 are equal, and have assumed that the errors made in the calculations of 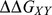 and 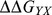 are uncorrelated. We then arrive at our estimates for the distribution of errors of the method
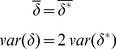
(7)


### Comparing methods

In order to compare methods, we would like to characterize the performance of these methods. A natural choice would be 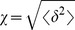, the root mean square of the error. Unfortunately, we can only compute statistics of 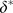, which as described above, gives an unbiased estimate of 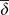 but underestimates the magnitude of 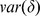 by a factor of 2. We can, however, rewrite
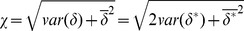
(8)


### Procedure

To create the dataset, all single chain PDB sequences were compared to each other and all pairs of sequences with only one amino acid change were selected. This provided 22947 pairs of proteins. To further reduce this number to a reasonable testing size and to allow for structural variability among the proteins, a pairs of proteins were randomly selected among SCOP (v1.75) families with a maximum of one pair from each family (although not all families are represented). [20]. This reduced the size of the dataset to 83 pairs of proteins. Further reduction of the dataset was done by removing pairs where the mutation was not resolved in the crystal structure (seven cases), pairs where either Rosetta (one case) or Eris (eight cases) could not read the PDB file, generally due to missing backbone atoms or unusual amino acid types, or when Eris produced either a failure notice or non-numerical output (two cases). This reduced the size of the dataset to 65 pairs of proteins which are listed in Table S1 along with the mutation made on each protein.

The Rosetta ddg_monomer program requires pre-minimized structures to remove possible clashes. Once the mutation is made, three iterations of the process were run starting with a lower repulsive value of the van der Waals term and increasing it to the normal value by the third round. This process allows for slight backbone movements in order to compensate for the side-chain substitutions. The minimization was done on both the wild type and mutated structures. To run the Rosetta ddg_monomer program, we used the recommended parameters finding the minimal 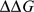 after fifty iterations of optimization [1]. FoldX was run based on recommendations from the authors. To obtain the 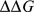 values from FoldX we ran the RepairPDB method to optimize the energy for each PDB file. We then ran the PositionScan method with the single point mutation to obtain the predicted values [5]. Eris was run on their web-server (http://dokhlab.unc.edu/tools/eris/index.html) using the recommended parameters allowing for flexible backbone and pre-relaxation of the structure [2] I-Mutant3.0 uses Support Vector Machine based predictors to obtain 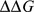 values from either a sequence or a structure. I-Mutant3.0 was ran using the structural option with standard set parameters for temperature and pH [8].

Confidence intervals were obtained through non-parametric bootstrapping. For each method, we generated a dataset of 65 pairs of homologous proteins by sampling our original set (with replacement), and calculated 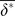, 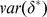, and 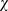. This was repeated 10,000 times. The fraction of these replicates where one method has a higher value of 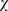 than another reflects the *P* value for the superiority of the first method. This approach was also used to indicate where the performance of a given method was statistically different on structure conserving mutations versus non conservative mutations, or for mutations at exposed versus buried locations.

The division of mutations into structure preserving and structure modifying sets was based on a calculation of the backbone atom RMSD between the two proteins in the pair; an RMSD cut-off of 0.4 gave us a set of 34 pairs of proteins for the low RMSD group and 31 pairs for the high RMSD group. The solvent accessibility was calculated using the Stride secondary structure classifier [21]. These values were normalized with the average solvent accessibility of each amino acid calculated by Oobatake, et al [22]. We then averaged the two RSA values for the protein pairs together to get the final RSA value. The buried group (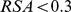) contains 32 pairs of proteins and the exposed group contains 33 pairs. The proteins that were used in both of these datasets can be found in Table S1.

In order to estimate the fraction of the variance explained by the different methods, we considered that the variance of the true values could be approximated by the RMS of the calculated values. Using this approximation, this estimate is equal to 
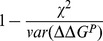
.

## Supporting Information

Figure S1
**A comparison of the bias and** 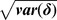 **values with the RMSD dataset scaled by RMS of the predictions.** The center bars represent the calculated value for each of the methods. The top and bottom bars represent the 67% confidence intervals and the thin vertical lines extend to the 95% confidence intervals. The order of methods is Rosetta (black), FoldX (red), Eris (green) and iMutant3.0 (blue). The open bars represent those pairs of proteins with small changes in the two structures (RMSD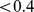) and the shaded bars represent the pairs with larger changes. For Eris, most of the difference between the datasets occurs in the bias. FoldX and I-Mutant3.0 have little change in bias with larger changes in the variance. Rosetta has small changes in both the bias and the variance.(EPS)

Figure S2
**A comparison of the bias and** 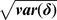 **values with the RSA dataset scaled by RMS of the predictions.** The center bars represent the calculated value for each of the methods. The top and bottom bars represent the 67% confidence intervals and the thin vertical lines extend to the 95% confidence intervals. The order of methods is Rosetta (black), FoldX (red), Eris (green) and iMutant3.0 (blue). The open bars represent those pairs of proteins with buried mutations (RSA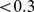) and the shaded bars represent the pairs with mutations that are more exposed. FoldX has the most differences in bias and variance than the others, likely due to a non-flexible backbone. I-Mutant3.0 has a larger bias in buried mutations, but a small change in the variance. Eris has little change in the bias but a large change in variance, and Rosetta has small changes in both.(EPS)

Table S1
**Raw data for each method.** The table contains the PDB id for the pairs of proteins, the mutation in each protein, the raw results (unscaled) for each of the methods in both directions, the SCOP fold, and which of the two groups for RMSD and RSA the proteins are in. The raw results are labeled with 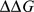 followed by the subscript for the method (R = Rosetta, F = FoldX, E = Eris, and I = I-mutant3.0). For the split, the 1 represents the proteins with RMSD
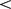
0.4 and the RSA
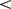
0.3. The mutation numbers are based on the residue number in the PDB file.(PDF)
