# Supplementary material for: Assessing Predictors of Changes in Protein Stability upon Mutation Using Self-Consistency
Source: PLoS One. 2012 Oct 29;7(10):e46084. doi: 10.1371/journal.pone.0046084 (PMC3483175; doi:10.1371/journal.pone.0046084)
Supplement: Table S1 — Raw data for each method. The table contains the PDB id for the pairs of proteins, the mutation in each protein, the raw results (unscaled) for each of the methods in both directions, the SCOP fold, and which of the two groups for RMSD and RSA the proteins are in. The raw results are labeled with followed by the subscript for the method (R = Rosetta, F = FoldX, E = Eris, and I = I-mutant3.0). For the split, the 1 represents the proteins with RMSD0.4 and the RSA0.3. The mutation numbers are based on the residue number in the PDB file. (PDF) [file pone.0046084.s003.pdf]

ID1	Mut	$\Delta\Delta G_R$	$\Delta\Delta G_F$	$\Delta\Delta G_E$	$\Delta\Delta G_I$	ID2	Mut	$\Delta\Delta G_R$	$\Delta\Delta G_F$	$\Delta\Delta G_E$	$\Delta\Delta G_I$	RMSD	RSA
2VM0	H64V	1.28	0.43	-1.86	-0.53	3HEP	V64H	1.46	1.81	2.62	1.78	2	1
1BEO	T6S	0.33	0.30	3.57	0.22	1LRI	S6T	-0.28	-0.05	0.04	-0.22	2	2
2FFN	Y65A	5.65	2.69	-6.75	1.77	2YYX	A65Y	-6.94	-2.02	-7.89	0.97	1	1
1ENK	D23E	-0.90	1.32	0.49	0.42	2END	E23D	2.31	0.95	2.17	0.37	1	1
1QPU	R98C	-0.53	0.20	-12.76	0.40	1QQ3	C98R	-3.18	-0.66	0.65	0.16	2	2
1CHH	Y82F	-0.91	-0.45	5.69	0.40	2YCC	F82Y	0.90	0.62	3.43	0.55	1	1
3CLN	N129D	-1.06	1.81	2.42	0.91	3IF7	D129N	1.46	-3.09	0.34	0.61	2	1
1A1W	Y25G	1.82	1.22	4.10	1.48	1AIZ	G25Y	-2.55	-0.97	-3.81	0.44	2	2
2YXF	W95L	4.62	0.10	-2.71	1.33	3QDA	L95W	-2.38	-0.10	0.67	1.96	2	1
1U3Z	C310M	-2.17	-1.46	1.26	0.02	1U42	M310C	2.71	0.03	2.05	0.36	2	2
2C9Q	H91F	-1.05	-0.88	-1.45	0.03	2C9R	F91H	5.00	0.82	0.05	1.43	2	2
1I5I	S18C	0.93	-0.29	-7.81	1.60	4GCR	C18S	-0.67	0.75	1.51	1.72	1	1
2CE6	N21D	-1.45	0.05	-3.47	0.40	2CGZ	D21N	-0.19	0.03	0.09	0.73	1	2
1UMH	C132A	-1.54	0.03	-0.20	0.98	1UMI	A132C	1.07	0.23	-0.31	0.88	2	1
1O9Z	S136A	1.19	-0.17	-1.53	1.18	3F6J	A136S	-0.13	0.76	1.05	0.82	1	2
2Q1M	N106A	0.88	0.43	0.16	0.07	2R30	A106N	-0.69	0.18	0.29	0.38	1	2
2NN8	P113A	0.11	-0.61	-4.39	0.62	2XG3	A113P	1.05	0.69	3.12	0.04	1	2
2P3I	R101A	3.31	2.46	1.22	1.26	3P3J	A101R	-1.61	-1.96	-4.53	0.42	1	1
2AU6	R43Q	-1.89	-0.28	-1.31	1.41	2AU7	Q43R	0.76	0.94	3.25	0.75	1	1
4FIV	Q99V	0.11	-0.44	1.28	0.14	6FIV	V99Q	-1.58	1.05	0.94	1.73	1	1
1UNR	E17K	0.26	0.68	2.74	0.90	2UZS	K17E	0.39	-0.44	1.99	0.44	2	2
3C78	E55Q	-1.46	-0.96	0.39	0.52	3FLL	Q55E	2.14	1.38	2.67	0.67	1	1
2CPL	R55K	0.23	-0.12	3.16	0.67	3K0R	K55R	0.29	0.06	-1.69	0.55	1	2
2PY4	H145A	1.68	0.14	-1.19	0.25	3LOJ	A145H	-1.53	-0.10	4.06	0.72	1	2
1Q92	D41N	-1.18	-2.43	-21.28	1.44	2JAW	N41D	1.15	3.03	1.71	1.59	1	1
5ULL	N137A	0.17	0.56	1.24	0.31	6ULL	A137N	-0.27	0.19	1.66	0.92	1	2
1M8F	A11K	-0.06	0.53	0.04	1.04	1M8G	K11A	0.21	0.95	-2.46	0.97	1	2
621P	H61L	1.26	-0.16	-3.13	-0.27	721P	L61H	-0.73	-0.02	0.46	1.03	1	2
1RXI	S82C	3.93	1.56	1.72	0.81	2FXI	C82S	-3.55	-0.09	4.65	0.74	2	1
1YS0	C473D	0.13	7.21	9.51	1.67	2IFV	D473C	5.08	1.63	2.19	1.37	2	1
2IIY	C73S	-1.40	0.10	2.65	0.26	2HSH	S73C	2.24	-0.66	-4.08	0.42	1	2
1O8X	A43C	3.94	1.68	1.13	0.98	1QK8	C43A	0.52	0.75	-3.61	1.25	1	1
2DVP	E152Q	-0.61	-0.06	3.36	0.08	2E5X	Q152E	0.64	0.03	-1.96	0.02	2	2
2RN2	A52F	-4.11	1.75	9.60	0.56	3AA5	F51A	6.38	3.96	6.73	2.48	2	1
1T2N	I157M	-0.56	-0.33	-2.94	0.88	3D2A	M157I	0.79	0.22	1.90	0.67	1	2
2ANQ	D37N	-1.06	-0.24	0.18	0.47	3DAU	N37D	1.25	0.36	2.29	0.05	2	2

1FY9	V223T	3.01	2.63	6.29	1.68	1FYA	T223V	3.82	3.11	3.60	0.98	2	1
7RNT	W45Y	-0.21	-0.92	-5.83	0.45	9RNT	Y45W	-1.829	0.73	6.09	-0.05	1	2
2D01	Y98Q	2.81	0.71	3.20	0.98	2I9V	Q98Y	-1.06	-0.22	-0.72	0.12	2	2
1V8T	E86Q	-1.40	-1.06	-0.86	0.55	1V8Y	Q86E	3.27	3.28	2.54	0.86	1	1
1EHB	F58Y	-0.79	0.93	-5.17	0.70	1U9U	Y58F	-0.28	-0.87	0.49	0.46	1	1
2RER	R69A	-0.88	4.01	4.32	1.59	2RES	A69R	3.37	-0.23	0.32	1.01	1	2
2FHI	N96H	-6.11	-0.34	-11.12	1.72	6FIT	H96N	5.04	1.42	2.54	0.88	2	1
1JVI	T96P	3.95	2.58	1.59	1.53	2FQT	P96T	5.45	5.19	9.26	1.56	1	1
1TT8	G90A	-2.88	-1.67	-2.76	1.09	1XLR	A90G	2.39	1.66	3.22	1.54	2	1
3LZT	E35A	0.06	0.59	1.96	0.46	3OK0	A35E	0.74	-0.21	0.70	0.75	2	1
2ESK	S94G	1.95	1.42	4.39	1.12	2ESQ	G94S	-0.79	-1.24	-1.52	1.39	2	2
2DJH	R33Q	1.14	2.25	1.36	1.07	3VJ7	Q33R	-1.62	-0.96	-2.30	0.42	1	1
2PPN	E60Q	-0.34	1.19	0.72	0.52	2PPP	Q60E	3.31	2.29	3.34	1.13	1	1
1DKH	H83A	3.34	0.12	0.80	0.14	2UYD	A83H	0.97	-1.62	1.85	0.39	2	2
3BZP	A262D	-0.84	0.65	-4.58	1.43	3BZS	D262A	1.54	-0.17	2.67	1.32	2	1
1LO8	D17N	-1.76	-0.40	-2.14	0.92	1LO9	N17D	1.50	0.17	1.49	0.55	1	1
1EOF	A136N	-0.42	-0.07	0.31	0.53	1T1D	N136A	0.24	0.18	-0.96	0.41	1	2
4RSD	A121D	0.69	1.17	1.35	1.06	9RAT	D121A	-0.19	0.37	1.88	1.01	2	2
2D27	L26V	0.67	0.38	0.94	0.81	2D28	V26L	-0.88	-0.45	-0.29	0.99	2	2
2FD2	A24C	3.17	0.26	0.10	1.26	7FDR	C24A	-2.57	0.34	-0.63	1.36	1	1
2I8E	A2M	1.00	-0.18	-1.09	0.22	2IVY	M3A	0.71	0.74	3.10	0.80	1	2
1EK8	R132G	1.16	2.06	5.10	1.69	1ISE	G132R	2.72	0.49	1.88	1.18	2	1
1BSW	A191S	0.51	0.25	4.91	0.73	1BUD	S191A	-1.27	-0.77	-0.30	0.77	1	2
1FU6	P75S	-0.56	1.84	-0.45	1.20	1OO3	S75P	6.97	0.83	1.53	0.19	2	2
1PQ0	F105A	8.76	4.45	5.90	1.51	3IIG	A105F	1.12	4.94	3.56	0.49	2	1
2O7L	S201T	0.76	0.88	-0.21	0.04	2XTV	T201S	0.19	1.39	0.53	0.29	2	1
1H2P	Q12E	0.34	0.28	0.46	0.09	1UOT	E12Q	1.67	-0.13	1.31	0.28	2	2
1CXX	A122R	0.90	-0.66	-0.34	0.76	1QLI	R122A	-0.45	-1.98	-0.35	1.41	2	2
3CP1	E71K	-0.67	-0.18	2.08	0.41	3CYO	K71E	-0.46	0.57	-1.22	0.37	1	2
